# Review of clinical practice guidelines on the diagnosis and treatment of third molars. Evaluation of adherence to AGREE II publication guideline

**DOI:** 10.4317/medoral.27578

**Published:** 2025-11-22

**Authors:** Mª Gemma Herráez-Tondo, Cosme Gay-Escoda, Jorge Toledano-Serrabona, Mª Àngels Sánchez-Garcés

**Affiliations:** 1 DDS. University of Barcelona, Barcelona (Spain); 2 DDS, MS, PhD. Senior Lecturer in Oral and Maxillofacial Surgery and Implant Dentistry at the University of Barcelona, Barcelona (Spain). Director of Master’s Degree Program in Oral Surgery and Implantology at EFHRE International University, Belize city (Belize). Researcher at the IDIBELL institute, Barcelona (Spain). Head of Integral Dentistry, Oral Surgery, Oral Implantology And Maxillofacial Surgery at Teknon Medical Center, Barcelona, Spain; 3 MD, DDS, PhD, Clinical assistant professor of Oral Surgery and Implant Dentistry. Master’s Degree Program in Oral Surgery and Implantology, School of Dentistry, University of Barcelona. Researcher at the IDIBELL institute, Barcelona (Spain); 4 MD, DDS, PhD, MS, EBOS, Associated professor of Oral Surgery and Implant Dentistry. Master’s Degree Program in Oral Surgery and Implantology, School of Dentistry, University of Barcelona. Researcher at the IDIBELL institute, Barcelona (Spain)

## Abstract

**Background:**

Clinical practice guidelines (CPG) are developed to summarize the available evidence for healthcare professionals and standardize decisions in the clinical setting. For them to be useful, they must be generated following systematic methods, have high scope and applicability, present information clearly, and be updated every five years. The main aim of this review was to evaluate the available CPGs on the diagnosis and treatment of third molars (3M) using the AGREE II instrument to assess their quality and strength of recommendations.

**Material and Methods:**

An electronic search was conducted using the PubMed (MEDLINE), Cochrane Library, and Scopus databases. Additionally, a manual search was performed by international health organizations and dental and surgical associations. The inclusion criterion was CPG published in the last 5 years on the diagnosis and treatment of 3M. The quality of the guidelines was analyzed using the AGREE II instrument.

**Results:**

14 CPGs were identified; However, only seven met the inclusion criteria. The guidelines from the Spanish Society of Oral Surgery (SECIB) and the Malaysia Oral Health Programme (MOHP) were considered high-quality. In contrast, guidelines from the German Medical Association (DGMKG), French Society of Stomatology Maxillo-Facial Surgery and Oral Surgery (SFSCMFCO) and Royal College of Surgeons of England (RCSE) were rated as moderate quality and recommended with modifications. The Finnish Medical and Dental Society (FMDS) and Dutch Association of Oral and Maxillofacial Surgeons (NVMKA) did not meet the minimum quality standards.

**Conclusions:**

The AGREE II analysis reveals a need for substantial improvement in third molar CPGs. Only two guidelines were rated as high-quality, with most being outdated or soon to be. Regular updates by guideline-developing organizations are essential to ensure accurate and relevant clinical recommendations.

## Introduction

The removal of third molars (3Ms) is one of the most common procedures in Oral Surgery. Third molars exhibit significant variability in development time, morphology, and the position of the crown and root. They are typically the last teeth to erupt in the oral cavity and may affect adjacent second molars (2Ms) by causing infectious, cysts, or tumors, among other complications. Impacted wisdom teeth are highly prevalent, occurring in 9% of cases in the upper jaw and 35% in the mandible. The cause of impaction can be attributed to a lack of space, an obstacle in the eruption path, or an inappropriate eruption direction (1 , 2).

The pathology associated with 3Ms is highly variable, which can create uncertainty regarding the appropriate professional approach. Due to ongoing scientific research and the high volume of published articles each year, there is a need to synthesize these findings to provide a source of information based on emerging evidence. Therefore, clinical practice guidelines (CPGs) are developed by different countries around the world (3 , 4). The main aim of CPGs is to establish specific protocols based on rigorous and updated scientific evidence, enabling professionals to make informed decisions and standardize the diagnosis and treatment of third molars. CPGs should be updated every five years, which is considered their period of validity (5).

The AGREE II is a valuable tool for assessing the content and quality of CPGs. It evaluates the methodological rigor and transparency of the guideline development process and provides a framework for achieving consensus on methodological standards and reporting criteria for international collaboration. The instrument consists of 23 items organized into six quality domains: Scope and purpose, stakeholder involvement, rigor of development, clarity of presentation, applicability, and editorial independence (6).

The main objective of this review was to evaluate the available CPGs for the diagnosis and treatment of third molars using the AGREE II instrument to establish their quality and grade of recommendation.

## Material and Methods

Search strategy

A PubMed-MEDLINE, Cochrane and Scopus databases search of articles was conducted between December 2023 and April 2025. The search algorithm in PubMed-MEDLINE was ("third molar"[All Fields] OR "third molar removal"[All Fields] OR "third molar extraction"[All Fields]) AND (consensus development conference [Filter] OR consensus development conference nih [Filter] OR guideline [Filter]).

The search algorithm in Scopus was (TITLE-ABS-KEY ("third molar" OR "third molar removal" OR "third molar extraction")) AND (DOCTYPE (cp) OR DOCTYPE (gu)) AND (LIMIT-TO ( SUBJAREA, "DENT")) AND ( LIMIT-TO ( EXACTKEYWORD, "Conference Paper"). The search algorithm in Cochrane was ("third molar" OR "third molar removal" OR "third molar extraction") AND (guideline OR consensus). Additionally, a manual search was performed through the main national and international health regulatory bodies, dental and surgical associations: National Institute for Health and Care Excellence (NICE), Guía Salud, Agency for Healthcare Research and Quality (AHRQ), Guidelines International Network (GIN), and Trip database. Furthermore, the references of the selected articles were examined. Grey literature was searched through OpenGrey (https://opengrey.eu).

Inclusion and exclusion criteria

The inclusion criteria were CPGs published in the last 5 years, which is their period of validity, addressing the diagnosis and treatment of 3Ms. All publications that did not meet the described inclusion criteria were excluded.

Analysis of Clinical Practice Guidelines

**Figure 1:**
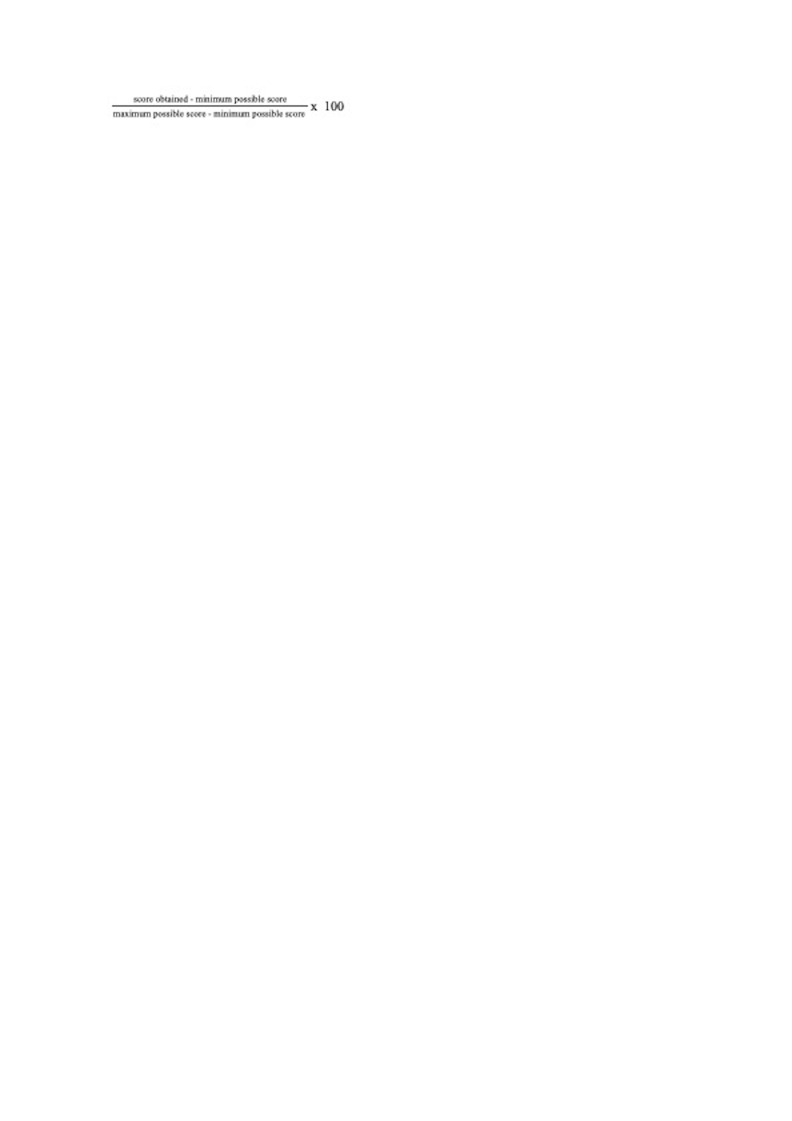
Percentage of achievement for each domain.

The CPGs were evaluated using the AGREE II instrument. Two independent evaluators (G.H-T and M.A. S-G.) assigned ratings on a scale from 1 to 7, where 1 indicates strong disagreement and 7 indicates strong agreement. Any discrepancies between the evaluators were resolved through discussion with a third researcher (J T-S). A quality score was calculated for each of the six domains defined by the AGREE II instrument. These domain scores are independent of each other. The percentage of achievement for each domain was calculated using the following formula (Figure 1):

1

The strengths and limitations of each CPG were identified, enabling a comparison of their methodological quality. To be considered high quality, a CPG must score above 75% in at least five domains. CPGs scoring 75% in four domains were classified as moderate quality, while those scoring below 75% in fewer than four domains were deemed low quality. After analysis, two overall evaluations were provided for each CPG: The first was an assessment of the guideline's quality based on the established criteria, and the second was a recommendation status, categorized as recommend, recommend with modifications, or not recommend.

## Results

**Figure 2:**
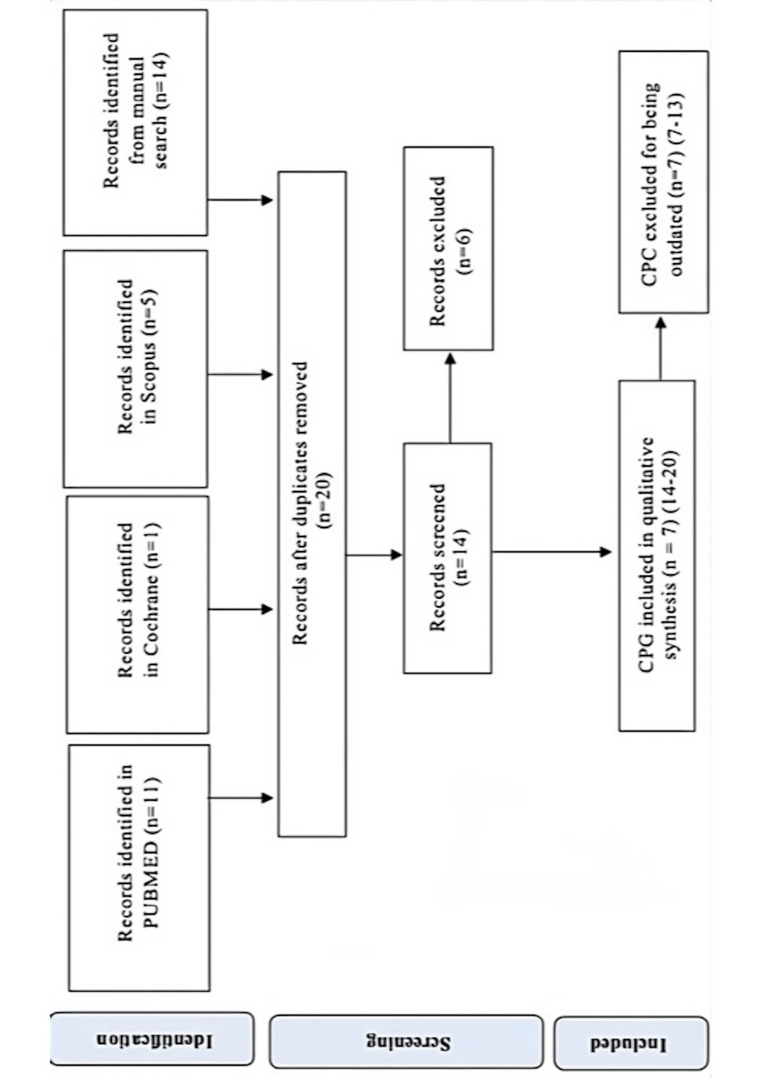
CPG selection process summarized flowchart.

Fourteen Clinical Practice Guidelines (CPGs) on the diagnosis and treatment of third molars (3Ms) were identified (Figure 2). However, half of these guidelines were outdated and were thus excluded from the analysis due to their lack of validity. Only seven CPGs met de inclusion criteria and were accepted for the analysis (7).

2

Scope and purpose

All CPGs describe their general objectives, except for the Dutch Association of Maxillofacial Surgeons (NVMKA). Health aspects are clearly described only in the guidelines from Malaysia Oral Health Programme (MOHP) and Spanish Society of Oral Surgery (SECIB). The Finnish Medical and Dental Society (FMDS) and French Society of Stomatology, Maxillo-Facial Surgery and Oral Surgery (SFSCMFCO) GPCs do not provide information on the target population but include details on the healthcare context and the interventions to be performed. The CPGs from FMDS, NVMKA, and Royal College of Surgeons of England (RCSE) do not specify the population to which the CPG is intended to apply. These aspects are reflected in the scores presented in Table 1.

Table 1

The analysis reveals significant variability in the application of the guidelines. DGMKG and MOHP guidelines provide directives for unerupted and/or impacted 3Ms. FMDS, NVMKA, and RCSE CPGs are only applicable to mandibular 3Ms. In contrast, the SFSCMFCO guideline offers an assessment of symptomatic versus asymptomatic 3Ms. Only the SECIB CPG includes recommendations for both upper and lower third molars, with or without pathology, symptomatic and asymptomatic.

Stakeholder involvement

To ensure the highest reliability of the Clinical Practice Guidelines (CPG), it is crucial that the members developing the recommendations provide their names, specialties, institutions, geographical locations, and a description of their roles in the development of the guidelines. The CPGs that include this information were those from DGMKG, SFSCMFCO, RCSE, MOHP, and SECIB.

In terms of clinical decision-making for 3Ms, patient preferences are particularly important in cases of asymptomatic 3Ms without pathology, where prophylactic extraction may be considered. Consequently, the CPGs from DGMKG, SFSCMFCO, RCSE, and SECIB have contemplated the patient's lifestyle and opinion.

Rigor of development

This section describes the procedure used to establish the recommendations. The appropriate method involves a systematic search, study selection, evaluation of the evidence, and assessment of the risk of bias. It also includes external review, which is necessary to verify scientific evidence, validate the justification of the recommendations, and obtain feedback on their clarity, providing an overall evaluation. Only two guidelines, MOHP and SECIB, specify that they were externally reviewed before publication. Furthermore, they are the only two guidelines that have been developed with the utmost methodological rigor.

Only DGMKG, MOHP, and SECIB guidelines establish a procedure for updating the guidelines.

Clarity of presentation

The recommendations are presented clearly, specifically, and without ambiguity in most of the CPGs. RCSE, MOHP, and SECIB guidelines stand out due to their clarity of presentation. The MOHP guideline includes an action algorithm for the management of unerupted and/or impacted 3Ms. On the other hand, the RCSE guideline provides a summary table on the clinical management of mandibular 3Ms.

Applicability

As shown in Table 1, this domain represents a weakness of all the guidelines except for SECIB and MOHP. The practical implementation of the guidelines, particularly the barriers and resource implications, varies significantly depending on the political context and the national health system. Despite this, only SECIB and MOHP guideline have addressed factors related to the applicability of the recommendations. The quality of the CPGs would increase substantially if the development groups addressed the issues encountered during implementation, such as organizational obstacles, economic impact, and dissemination strategies.

Editorial independence

To implement a healthcare protocol, it is essential to ensure the objectivity and transparency of the CPGs through the editorial independence of the authors. In this regard, the guidelines from DGMKG, SFSCMFCO, RCSE, MOHP, and SECIB have recorded and addressed conflicts of interest, ensuring that the viewpoints of the funding entity did not influence the content of the guidelines. However, the CPGs from FMDS and NVMKA have not documented this aspect.

Recommendation grade

According to the results shown in Table 1, the recommendations of SECIB and MOHP guidelines are established with a high degree of quality. Conversely, the guidelines from RCSE, SFSCMFCO, and DGMKG require modifications to achieve the necessary level of reliability before they can be recommended. In contrast, the FMDS and NVMKA guidelines do not meet the minimum quality requirements to be recommended according to the AGREE II criteria.

## Discussion

This review analyzed the quality of Clinical Practice Guidelines (CPGs) for the diagnosis and treatment of third molars using the AGREE II instrument. The findings suggest that most of the assessed guidelines have notable areas in need of enhancement, particularly in the areas of methodological rigor and applicability. Only the guidelines from the SECIB and the MOHP were rated as high quality, demonstrating thorough development processes and clear recommendations. In contrast, other guidelines, including those from the DGMKG and the RCSE, require modifications to meet acceptable standards.

In 1999, the Scottish Intercollegiate Guidelines Network (SIGN) published a CPG (8) that advised against the prophylactic treatment of 3Ms due to insufficient supporting evidence. Similarly, the guideline from the National Institute for Health and Care Excellence (NICE) (9), published in 2000, significantly impacted decision-making regarding the extraction of 3Ms. This guideline also highlighted the lack of evidence for prophylactic extraction, suggesting that clinical indications for wisdom tooth extraction should be limited to patients with associated disease. It has been demonstrated that the publication of these CPGs influenced the behavior of health professionals, as the number of 3M extractions without pathology significantly decreased following the publication of these guidelines (10). Therefore, it is evident that CPGs play a fundamental role in the clinical judgment of professionals and have a substantial impact on decision-making.

However, the measures established by SIGN (8) and NICE (9), instead of reducing the number of third molar (3M) extractions, caused a demographic shift in surgical interventions in the United Kingdom. Specifically, McArdle and Renton (10) observed that the average age of patients increased from 25 years to 32 years after the implementation of NICE's guidelines. Additionally, the adoption of these guidelines also led to an increase in the number of extractions of 3Ms with pathology (11). Thus, dental clinicians faced older patients and more cases of 3Ms with pathology, which increased the cost of 3M management for the National Health System in the United Kingdom.

Current evidence indicates that asymptomatic 3Ms rarely remain disease-free. The most frequent pathologies associated with 3Ms and/or adjacent second molars (2Ms) are caries and periodontal disease (12). The primary cause of these conditions is plaque accumulation, which occurs due to difficulties in maintaining adequate oral hygiene in the posterior regions of the mouth. This situation can worsen when the 3M is partially erupted, as it disrupts the gingival seal and creates a pathway between the tooth and the oral cavity. Furthermore, the tissue located distal to the 3M has usually unfavorable periodontal properties, as it consists of mucosa from the tonsillar pillar rather than attached gingiva (12 - 14).

Regarding the management of 3Ms, the absence of symptoms does not imply the absence of pathology (15 , 16). Therefore, in cases where the 3M is in a mesioangular, horizontal, or partially erupted position, prophylactic extraction is increasingly being recommended (12 , 15 - 21). Bouloux et al. (22) conducted a study on patients with asymptomatic 3Ms, finding cumulative incidence rates for 3M extraction ranging from 5% per year to 64% over 18 years. Thus, the cumulative risk of extracting asymptomatic 3Ms over time versus the risk of possible peri- and postoperative complications should be considered when informing the patient and establishing the most appropriate treatment plan. If the benefits of extraction outweigh the risks of retaining the 3M, surgical intervention at the earliest possible age is indicated (22). Conversely, for cases where 3Ms are retained, patients must undergo active clinical and radiographic monitoring during periodic follow-up visits. Despite evidence suggesting that most patients will eventually require 3M extraction, some may retain them throughout their lives. Therefore, it is essential to monitor these patients and maintain an attitude of active surveillance (7 , 22).

Current data suggest that as patients age, 3M extraction becomes more complex, the duration of the intervention increases, and postoperative recovery becomes longer and less complete. Additionally, the risk of complications rises with age (23 - 25).

The economic and political aspects of each country should be considered in the implementation of recommendations. This is reflected in the National Health Service (NHS), which opposes prophylactic extraction (9). This contraindication may be influenced by increasing pressure to reduce costs in the NHS, leading to a strictly therapeutic approach to 3M surgery. In contrast, the 2007 guidelines of the American Association of Oral and Maxillofacial Surgeons (AAOMS) included a more prophylactic interventionist approach, possibly indicating support for private healthcare surgical interventions. However, in 2016, the AAOMS guidelines were revised, introducing a change in approach based on new evidence, and recommending active surveillance (5). This demonstrates the importance of evaluating emerging high-quality evidence and periodically updating guidelines.

The process of developing guidelines requires economic resources and a significant investment of time. CPGs should be updated every 3-5 years; those published with more than 5 years of validity are considered outdated. Therefore, creating a way to unify criteria and establish collaborations between associations from different countries would promote efficient use of resources and could lead to homogenization in decision-making.

While this review provides a comprehensive analysis of CPGs for the diagnosis and treatment of third molars using the AGREE II instrument, several limitations should be noted. First, the inclusion criteria restricted the analysis to CPGs published within the last five years, which may have excluded relevant older guidelines that could still offer valuable insights. Second, the assessment was based only on the information provided within the CPGs; thus, any additional unpublished data or context that could affect the guidelines' applicability and accuracy was not considered. Third, the reliance on the AGREE II instrument, although comprehensive, may not fully capture all dimensions of guideline quality, especially those related to specific clinical contexts and cultural variations in medical practice. Lastly, there may be inherent biases in the CPGs included, as they are developed by specific organizations with particular interests, which could influence the recommendations. These limitations highlight the need for continuous updates and critical appraisal of CPGs, as well as the importance of considering a broader range of evidence and contexts in future analyses.

## Conclusions

The quality analysis of Clinical Practice Guidelines using the AGREE II instrument reveals a need for significant improvements in the development processes of some guidelines. It is essential for guideline development organizations to update their recommendations regularly, following AGREE II standards, to maintain accurate and relevant clinical practice information. The analysis also highlights the potential benefits of international cooperation among scientific organizations, which could enhance efficiency and resource utilization, ultimately aiming for excellence in the diagnosis and treatment of third molars.

## Figures and Tables

**Table 1:** Table Results of the analysis using AGREE II. Classification into High, Moderate, or Low quality is based on the number of domains achieving a score of 75% or higher. High-quality CPGs had at least 5 domains with 75%, Moderate-quality CPGs had 4 domains with 75% and Low-quality CPGs had fewer than 4 domains with 75%.

	Scope and purpose	Stakeholder involvement	Rigor of development	Clarity of presentation	Applicability	Editorial independence	Quality
DGMKG (1)	94.44%	94.44%	58.33%	94.44%	70.83%	100%	Moderate
FMDS (2)	55.55%	66.67%	25%	88.89%	54.16%	0%	Low
SFSCMFCO (3)	77.78%	100%	58.33%	88.89%	50%	100%	Moderate
NVMKA (4)	11.11%	50%	14.58%	83.33%	12.5%	0%	Low
RCSE (5)	66.7%	100%	75%	100%	50%	100%	Moderate
MOHP (6)	100%	66.7%	100%	100%	83%	100%	High
SECIB (7)	100%	100%	100%	100%	100%	100%	High

DGMKG: German Medical Association, FMDS: Finnish Medical and Dental Society, SFSCMFCO: French Society of Stomatology, Maxillo-Facial Surgery and Oral Surgery, NVMKA: Dutch Association of Oral and Maxillofacial Surgeons, RCSE: Royal College of Surgeons of England, MOHP: Malaysia Oral Health Programme, SECIB: Spanish Society of Oral Surgery.

## Data Availability

The datasets used and/or analyzed during the current study are available from the corresponding author.
